# Effects of COVID-19 on Irish general practice activity from 2019 to 2021: a retrospective analysis of 500,000 consultations using electronic medical record data

**DOI:** 10.1007/s11845-024-03810-6

**Published:** 2024-10-02

**Authors:** Michael E. O’Callaghan, Liam G. Glynn

**Affiliations:** 1 School of Medicine, North Campus, University of Limerick, Limerick, Ireland; 2 Irish College of GPs, 4/5 Lincoln Place, Dublin 2, Ireland; 3 Health Research Institute, University of Limerick, Limerick, Ireland

**Keywords:** COVID-19, Electronic medical records, General practice, Primary care, Teleconsultation

## Abstract

**Background:**

General practice (GP) is crucial to primary care delivery in the Republic of Ireland and is almost fully computerised. General practice teams were the first point of contact for much COVID-19-related care and there were concerns routine healthcare activities could be disrupted due to COVID-19 and related restrictions.

**Aims:**

The study aimed to assess effects of the pandemic on GP activity through analysis of electronic medical record data from general practice clinics in the Irish Midwest.

**Methods:**

A retrospective, descriptive study of electronic medical record data relating to patient record updates, appointments and medications prescribed across 10 GP clinics over the period 2019–2021 inclusive.

**Results:**

Data relating to 1.18 million record transactions for 32 k patients were analysed. Over 500 k appointments were examined, and demographic trends presented. Overall appointment and prescribing activity increased over the study period, while a dip was observed immediately after the pandemic’s arrival in March 2020. Delivery of non-childhood immunisations increased sixfold as a result of COVID-19, childhood immunisation activity was maintained, while cervical smears decreased in 2020 as the screening programme was halted. A quarter of consultations in 2020 and 2021 were teleconsultations, and these were more commonplace for younger patients.

**Conclusions:**

General practice responded robustly to the pandemic by taking on additional activities while maintaining routine services where possible. The shift to teleconsulting was a significant change in workflow. Analysing routinely collected electronic medical record data can provide valuable insights for service planning, and access to these insights would be beneficial for future pandemic responses.

## Introduction

Primary care addresses the main health problems in communities, providing “promotive, preventive, curative, and rehabilitative services accordingly” [1]. In the Republic of Ireland, general practice teams are critical to primary care provision, providing an average of four GP consultations to each person in the state each year [2]. Almost all Irish general practitioners (GPs) have computerised their practices [3], with practices using one of four accredited electronic medical record (EMR) programmes to deliver care to patients [4]. The leading package is “Socrates”, used by more than 55% of GPs [5].

Preventive care provided routinely by Irish general practice teams includes the primary childhood immunisation programme (PCIP), which involves all vaccinations delivered in the first years of life [6, 7] and health screening via state-supported programmes such as the cervical screening programme “Cervical Check” [8].

The COVID-19 pandemic disrupted provision of routine healthcare due to a combination of demand-side issues, such as patient avoidance of healthcare settings due to fear of exposure and fear of burdening health systems; and supply-side issues, including service cancellations, staff redeployment, facility closures, and supply chain problems [9, 10]. As the virus spread and hospitals braced for the potential impact, opportunities for routine healthcare provision in primary care were disrupted [11] and concerns were expressed regarding knock-on effects of such missed opportunities [12, 13], including vaccination programmes and cancer screening.

This project aims to assess the effects of the pandemic on routine GP care activities by analysing summary electronic medical record data relating to patient record updates, appointments and medications prescribed.

## Methods

This study used anonymized retrospective visit data from GP EMRs. Ethical approval was granted through the University of Limerick Hospital Group Research Ethics Committee (Reference 069/2021, approved 11th June 2021).

### Setting

All thirty practices of the ULEARN-GP Research and Education network [14] in the Irish Midwest (counties Limerick, Clare and North Tipperary) using the Socrates EMR were invited to participate in this study by email. With a broad spread of rural and urban areas [15] and population of over 400 k (8% of national total), the Midwest is broadly representative of the national picture, though has a slightly older and more deprived population than that seen nationally [16]. Ten daytime general practice services (2 city, 2 large town, 4 small town and 2 rural practices) agreed to participate.

### Data collection

Summary data pertaining to all entries to patient files, all patient appointments and all medicines prescribed during the 3 years 2019 to 2021 inclusive were collected. Standard reports available through the software were used to provide raw data for the study period. All data were anonymised irrevocably on site using custom software written by the lead author to process reports from the practice EMR. Anonymised data were sent back to the research team using the Health Service Executive secure email service “Healthmail”.

### Analyses

Descriptive statistics using standard formulae and analyses were carried out using the open source statistical software package R [17] to assess trends in overall prescribing and overall practice activity over the study period, exploring in particular the provision of telephone consultations, childhood immunisations and cervical screening tests. Differences in proportions of consultation types from 2019 versus 2021 and in patients receiving telephone consultations versus all appointments by age group were assessed using the χ^2^ test.

## Results

There were 1.18 million entries made to 31.9 k patient records in the ten practices over the study period, which translates as just over 1 entry per chart per month for every patient in the dataset. More than half of these entries were tasks or updates to records completed by the various general practice team members over the course of the normal working day. Of the total number of entries, 510 k were recorded as distinct appointments delivered by GPs, practice nurses and other healthcare professionals. Of these 510 k appointments, 1% were completed by GP interns (first year post graduation), 5% by GP registrars (GPs in training), 30% by practice nurses and 61% by GPs, with the remaining 2.5% of entries completed by other ancillary health professionals and students. Numbers of healthcare professionals working in the practices was similar across the study period. Across the ten practices, there were 376 distinct consultation types relating to appointments provided over the 3 years, which were classified into the eight Consultation type categories shown in Table 1.

**Table 1:** Overall appointment activity over the 3-year study period across 10 general practice clinics in the Midwest of Ireland, classified by consultation type. The right-most column examines for significant changes in proportions of the various consultation types from 2019 versus 2021

Consultation type	2019	(%)	2020	(%)	2021	(%)	Total (3 years)	2019 vs 2021 ${\text{X}}^{2}$ test
1- **General**	136,674	88%	90,035	57%	97,835	50%	**324,544**	*
2- **Teleconsultation**	2876	2%	39,506	25%	44,788	23%	**87,170**	*
3- **Prescribing**	5372	3%	12,787	8%	17,323	9%	**35,482**	*
4- Vaccine (flu/PCV**/COVID)	2707	2%	6671	4%	22,706	12%	**32,084**	*
5- **Admin**	1116	1%	3677	2%	5932	3%	**10,725**	*
6- **Clinical admin**	2669	2%	2873	2%	3902	2%	**9444**	*
7- **Vaccine (childhood)**	1930	1%	2303	1%	2231	1%	**6464**	*
8- **Cervical screening test**	1228	1%	1015	1%	2187	1%	**4430**	*
**Total** **(Consultation types)**	**154,572**		**158,867**		**196,904**		**510,343**	*p < 0.01

^**^PCV, pneumococcal vaccine (adult)

**Fig. 1:**
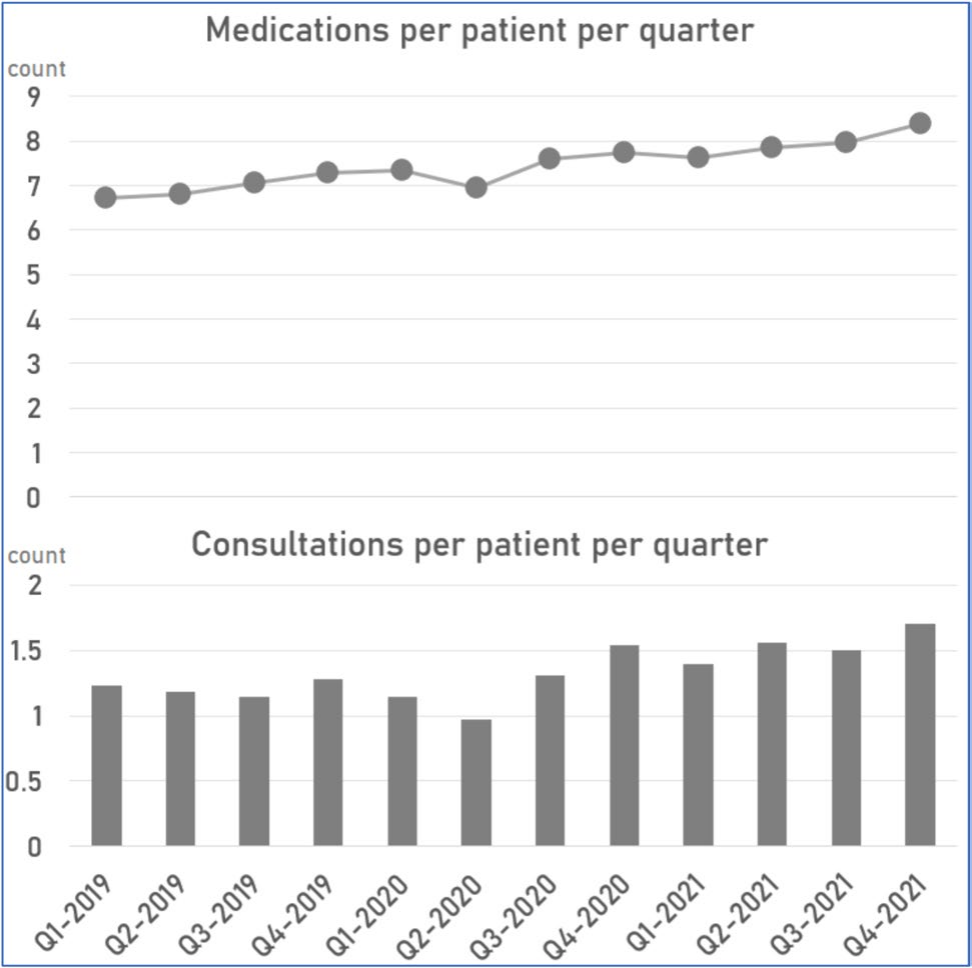
Breakdown of appointment and prescribing activity per patient per quarter

There were 2.85 million medication items prescribed by the ten practices over the study period, which if taken across the study population of 31.9 k patients translates as just under 2.5 items per patient per month (or 7.5 items per quarter) over the study period. Figure 1 shows the breakdown of appointment and prescribing activity per patient per quarter.

For average appointment count per patient by age group in the dataset for 2021, there were 3.7 appointments per patient aged 0–17 years, 5.4 appointments per patient aged 18–44 years, 6.4 appointments per patient aged 45–64 years and 10.8 appointments per patient aged 65 years-plus in 2021.

Finally, Table 2 demonstrates the breakdown of practice recording of main appointment types in 2021. For the differing age ranges in the left-most column of Table 2, the proportion of in each age grouping receiving care via teleconsultation was assessed using the χ^2^ test, yielding significant results (*p* < 0.01).

**Table 2:** Breakdown of activity (general, teleconsultation, other) by practice for 2021

**2021 data**													
**Age range**	Consultation type	**Practice A**	**Practice B**	**Practice C**	**Practice D**	**Practice E**	**Practice F**	**Practice G**	**Practice H**	**Practice I**	**Practice J**	**Total**	
**All ages**	**Total**	58,178	15,631	17,033	20,955	8959	14,859	6337	20,761	17,077	17,114	196,904	**Proportion tele-consults to total by age range**
	General	23,725	8798	13,235	7669	3469	6632	2018	14,932	10,093	7264	97,835	
	Teleconsultation	22,014	1757	229	4017	2819	1695	2483	3056	4266	2452	44,788	
	Other˄	12,439	5076	3569	9269	2671	6532	1836	2773	2718	7398	54,281	
**0–17 years**	General	2612	561	1463	662	453	883	267	1439	1034	579	9953	27.3%*
	Teleconsultation	3057	111	129	345	174	237	262	336	656	346	5653	
	Other˄	1331	230	444	729	445	586	174	333	417	429	5118	
**18–44 years**	General	7002	1636	3723	1795	1122	2320	604	3611	2947	2082	26,842	26.0%*
	Teleconsultation	7417	464	50	1123	1183	628	772	1000	1276	835	14,748	
	Other˄	4325	655	987	2405	887	2351	336	1051	488	1683	15,168	
**45–64 years**	General	6673	2264	3686	1942	972	1214	412	4056	2709	2030	25,958	23.9%*
	Teleconsultation	6191	469	34	1060	846	332	530	784	1058	600	11,904	
	Other˄	3448	1346	321	2303	564	1103	322	405	270	1897	11,979	
**65 years + **	General	7352	4325	4355	3264	916	2192	735	5819	3380	2471	34,809	18.0%*
	Teleconsultation	5320	713	16	1489	616	496	919	936	1268	645	12,418	
	Other˄	3321	2845	1817	3832	771	2490	1004	984	1543	3322	21,929	
**Unknown**	General	86	12	8	6	6	23	0	7	23	102	273	15.3% *p < 0.01 ${\text{X}}^{2}$ test
	Teleconsultation	29	0	0	0	0	2	0	0	8	26	65	
	Other˄	14	0	0	0	4	2	0	0	0	67	87	
	**Total**	58,178	15,631	17,033	20,955	8959	14,859	6337	20,761	17,077	17,114	196,904	

˄Other = prescribing, vaccines (flu/pneumococcal/COVID and childhood), admin, clinical admin, cervical screening test

## Discussion

### Summary of main findings

This study demonstrates that general practice activity increased from 2019 to 2021, with particularly large increases in teleconsulting and vaccination activity, likely driven directly by COVID-19. In addition, activity levels in cancer screening (when permitted again in 2021) and childhood vaccination programmes were not adversely affected by the pandemic. General practice continued to deliver these essential preventative activities. There was a notable shift towards more granular recording of activity and while lack of standardisation across practices introduced some analysis challenges, grouping of activity facilitated comparisons between practices and over time. Over the course of the study period, delivery of GP care for 31.9 k patients involved provision of 510 k consultations recorded in the EMR but involved more than twice this number of record entries in patient charts by GP teams. This speaks to the hidden workload of general practice that is not readily assessed by standard metrics, which tend to focus on consultation numbers [18], which will miss a large portion of work required to deliver general practice-based care [19, 20].

### Comparison with existing literature

General practice care delivery in Ireland is a team effort, with nurses being responsible for almost a third of appointment activity in the study, with GPs and GPs in training being responsible for approximately two-thirds, which is in keeping with national estimates [5]. As may be expected, patients at extremes of age have increased care needs [2]. While COVID-19-related illness, long-COVID and COVID-19 vaccinations have all been drivers of increased GP workload in Ireland in recent years [21, 22], demographic trends leading to a higher proportion of patients in older age groups also continue to exert demand side pressures on general practice [23].

Overall, consulting activity reduced for a period in early-mid 2020, before rebounding in 2021 (see Fig. 1). Prescribing activity did not dip to the same degree, and indeed is in keeping with the generally upwards trend seen nationally [22]. Growth of teleconsulting, on top of the pre-pandemic consultation workload, means that GP teams are likely now busier than ever before.

Adapting to workload challenges and pandemic pressures through use of teleconsulting seems apparent in our study, although the pre-pandemic picture seems to be incomplete. Just 2% of activity in 2019 was recorded as teleconsultations, which is much lower than the 10.5% reported elsewhere [24]. It is felt that better recording of this workstream occurred as GP teams adapted appointment booking systems and their workflow to cope with additional demand for teleconsultations. This adaptation led to teleconsultation appointments being recorded more faithfully as GPs diverted some of their time to dealing with this substantial shift in workload. National data suggests that teleconsulting activity is falling, with 60% of GP consults in October 2020–March 2021 (a period of very high COVID-19 activity) being delivered face-to-face, compared to 84% in early 2022 [25, 26]. This may indicate patient and/or GP preference for face-to-face consults [27, 28].

The higher likelihood of teleconsultations being used for younger age groups is perhaps expected given their increased levels of comfort with technology and less complex overall care needs.

In any case, consequences of the dramatic increase in recorded teleconsultation activity for overall workload and unintended consequences require further study. While potential to increase inequality of care provision to already disadvantaged groups [29], medicolegal uncertainty for care providers [30] and uncertainty around impact on antimicrobial stewardship [31] mean we should tread carefully, it is perhaps the potential to undermine the “establishment and maintenance of relational trust, with a negative impact on the quality of care and patient safety” [32] that requires most consideration. Interestingly, patients seem to value teleconsultation with the primary care physician they know well [33], which highlights the existing relationship we have with patients, often formed over years, as the foundation for high-quality and effective care [34].

Regarding provision of routine care, childhood vaccinations increased over the course of the pandemic, which is in keeping with national trends [35], and may be important given recent measles outbreaks across Europe [36]. Cervical screening tests dipped in 2020 before rebounding in 2021, which is in keeping with a national closure of the programme for 3 months in 2020 [37].

### Strengths and limitations

This analysis of this large body of real-world data offers an overview of general practice activity in the Midwest of Ireland over the study period. Analysis of all EMR entries, in addition to prescribing and consulting activity, highlights the “hidden workload” relating to administrative and follow-up tasks that is critical to well-functioning primary care.

Recruitment via email to a University-associated research network may introduce selection bias, and generalisability may be adversely affected by the selection of one specific EMR and the Irish Midwest as our study setting. Incomplete or inaccurately recorded EMR data could also affect validity. Finally, consequences stemming from teleconsultations (e.g. did a teleconsultation lead to a prescription or a future appointment?) were not recorded in this study.

## Conclusions

As seen internationally [38], general practice in Ireland was challenged and changed by the pandemic, adapting swiftly despite resource constraints and maintaining care provision where permitted. Activity is now being recorded more faithfully by practices, particularly for teleconsultations, and while evidence around teleconsulting’s long-term benefit is lacking, this element of GP workflow seems to be here to stay [2].

Study of GP EMRs can offer useful insights into workflow trends and while standardisation of recording activity is desirable, we must avoid burdensome EMR activities, such as excessive data entry or administrative work that is not valuable to the clinician or the patient [39, 40], as this may interfere with direct patient care and contribute to physician burnout [41].

GP EMR data can validate data from other sources [35], while providing more granular information relating to patient and healthcare professional characteristics, to aid timely assessment of immediate challenges such as pandemics and long-term planning in the face of changing population and demographic trends.

## Abbreviations

EMR: Electronic medical record
GP: General practice / General practitioner
COVID-19: Coronavirus disease 2019
PCIP: Primary childhood immunisation programme
U-LEARN: University of Limerick Education and Research Network

## References

[1] Bryant JH, Richmond JB (2017) Alma-Ata and primary health care: an evolving story. In: Quah SR, editor. International Encyclopedia of Public Health (Second Edition) [Internet]. Oxford: Academic Press; 2017 [cited 2024 Feb 14]. p. 83–102. Available from: https://www.sciencedirect.com/science/article/pii/B9780128036785000175

[2] DOH Ireland. Healthy Ireland Survey 2023 [Internet]. 2023 [cited 2024 Jan 16]. Available from: https://www.gov.ie/en/publication/73c9d-healthy-ireland-survey-2023/

[3] O’Kelly M, Teljeur C, O’Kelly F, Shuilleabhain AN, O Dowd T. Structure of general practice in Ireland 1982–2015 [Internet]. 2016. Available from: https://www.tcd.ie/media/tcd/medicine/public-health-primary-care/pdfs/structure-of-general-practice-2016.pdf

[4] ICGP. Software Companies - ICGP Web Site [Internet]. 2018 [cited 2024 Mar 2]. Available from: https://www.icgp.ie/go/in_the_practice/it_in_the_practice/software_companies

[5] ICGP (2021) Irish College of General Practitioners- Professional Competence Scheme data 2021/22

[6] Immunisation Schedule (2024) [Internet]. HSE.ie. [cited 2024 Feb 11]. Available from: https://www.hse.ie/eng/health/immunisation/pubinfo/pcischedule/immschedule/immunisation.schedule.html

[7] HSE National Immunisation Office (2016) Guidelines for vaccinations in general practice [Internet]. 2016 Sep. Available from: https://www.icgp.ie/speck/properties/asset/asset.cfm?type=LibraryAsset&id=3FFD799A%2DF3E4%2D5C77%2D0668C7DE68901A5F&property=asset&revision=tip&disposition=inline&app=icgp&filename=guidelinesGP%2Epdf

[8] National Screening Service (2021) Cervical Check Programme Report September 2017-March 2020 [Internet]. 2021. Available from: https://assets.hse.ie/media/documents/CervicalCheck_Programme_Report_2017-2020.pdf

[9] WHO. Pulse survey on continuity of essential health services during the COVID-19 pandemic: interim report, 27 August 2020 [Internet]. 2020 Aug [cited 2024 Feb 14]. Available from: https://www.who.int/publications-detail-redirect/WHO-2019-nCoV-EHS_continuity-survey-2020.1

[10] Einav S, Tankel J (2022) The unseen pandemic: treatment delays and loss to follow-up due to fear of COVID. J Anesth Analg Crit Care 2(1):5 37386539 10.1186/s44158-021-00032-5 PMC8795953

[11] Strengthening the frontline: how primary health care helps health systems adapt during the COVID 19 pandemic [Internet]. OECD. [cited 2024 Feb 14]. Available from: https://www.oecd.org/coronavirus/policy-responses/strengthening-the-frontline-how-primary-health-care-helps-health-systems-adapt-during-the-covid-19-pandemic-9a5ae6da/

[12] Vellinga A, Mellotte M, Mealy PJ et al (2022) Corona citizens’ science project-repeated surveys of the Irish response to COVID-19 and subsequent lockdown and restrictive measures. Ir J Med Sci 191(2):577–588 33761094 10.1007/s11845-021-02582-7 PMC7988378

[13] Kennelly B, O’Callaghan M, Coughlan D et al (2020) The COVID-19 pandemic in Ireland: an overview of the health service and economic policy response. Health Policy Technol 9(4):419–429 32923355 10.1016/j.hlpt.2020.08.021 PMC7480279

[14] O’Regan A, Hayes P, O’Connor R et al (2020) The University of Limerick Education and Research Network for General Practice (ULEARN-GP): practice characteristics and general practitioner perspectives. BMC Fam Pract 21(1):25 32024480 10.1186/s12875-020-1100-y PMC7003418

[15] Introduction urban and rural life in Ireland, 2019 - Central Statistics Office [Internet]. CSO; [cited 2024 Sep 12]. Available from: https://www.cso.ie/en/releasesandpublications/ep/p-urli/urbanandrurallifeinireland2019/introduction/

[16] Health Service Executive (HSE). Regional population profile - health region: Mid-West [Internet]. 2024 Mar. Available from: https://www.hse.ie/eng/about/who/healthwellbeing/knowledge-management/health-intelligence-files/hr-mid-west-profile-census-2022.pdf

[17] R Core Team. R: a language and environment for statistical computing. [Internet]. R Foundation for Statistical Computing, Vienna, Austria.; 2021. (R Foundation for Statistical Computing, Vienna, Austria.). Available from: https://www.R-project.org/

[18] Woolford SJ, Watson J, Reeve J, Harris T (2024) The real work of general practice: understanding our hidden workload. Br J Gen Pract 74(742):196–197 38664043 10.3399/bjgp24X737061 PMC11060809

[19] Crosbie B, O’Callaghan ME, O’Flanagan S et al (2020) A real-time measurement of general practice workload in the Republic of Ireland: a prospective study. Br J Gen Pract 70(696):e489–e496 32482628 10.3399/bjgp20X710429 PMC7274543

[20] Sinnott C, Moxey JM, Marjanovic S et al (2022) Identifying how GPs spend their time and the obstacles they face: a mixed-methods study. Br J Gen Pract 72(715):e148–e160 34844920 10.3399/BJGP.2021.0357 PMC8813099

[21] DOH Ireland. COVID-19 HSE Weekly Vaccination Figures - data.gov.ie [Internet]. [cited 2024 Jan 15]. Available from: https://data.gov.ie/dataset/covid-19-hse-weekly-vaccination-figures1

[22] PCRS. PCRS - Reporting Menu [Internet]. Annual Report 2022. 2022 [cited 2024 Jan 19]. Available from: https://www.sspcrs.ie/portal/annual-reporting/report/annual

[23] CSO. Vital Statistics Yearly Summary 2021 - CSO - Central Statistics Office [Internet]. CSO; 2022 [cited 2024 Jan 27]. Available from: https://www.cso.ie/en/releasesandpublications/ep/p-vsys/vitalstatisticsyearlysummary2021/

[24] Homeniuk R, Collins C (2021) How COVID-19 has affected general practice consultations and income: general practitioner cross-sectional population survey evidence from Ireland. BMJ Open 11(4):e044685 38607944 10.1136/bmjopen-2020-044685 PMC8039245

[25] DOH Ireland. Healthy Ireland Survey 2021 [Internet]. 2021 [cited 2024 Jan 16]. Available from: https://www.gov.ie/en/publication/9ef45-the-healthy-ireland-survey-2021/

[26] DOH Ireland. Healthy Ireland Survey 2022 [Internet]. 2022 [cited 2024 Jan 16]. Available from: https://www.gov.ie/pdf/?file=https://assets.gov.ie/241111/e31b2aaa-a8d7-411d-8b62-02cca079c741.pdf#page=null

[27] Goode C, Manorekang R, Durham N. Do patients prefer face-to-face or telephone consultations in an outpatient cardiology department? Eur Heart J - Digit Health. 2022 Dec 22;3(4):ztac076.2819.

[28] Antonio S, Joseph D, Parsons J, Atherton H (2024) Experiences of remote consultation in UK primary care for patients with mental health conditions: a systematic review. Digit Health 1(10):20552076241233970 10.1177/20552076241233969 PMC10924560 38465292

[29] Stewart M, Brown J, Weston, WW, Freeman T, McWhinney I (2024) Patient-centered medicine transforming the clinical method [Internet]. 4th ed. Chapter 9- Patient-Centered Approaches in the Face of New Technologies; 2024 [cited 2024 Mar 16]. Available from: https://www.taylorfrancis.com/chapters/edit/10.1201/9781003394679-12/patient-centered-approaches-face-new-technologies-moira-stewart-bridget-ryan-thomas-freeman

[30] Fernández Coves A, Yeung KHT, van der Putten IM, Nelson EAS (2022) Teleconsultation adoption since COVID-19: comparison of barriers and facilitators in primary care settings in Hong Kong and the Netherlands. Health Policy 126(10):933–944 36050194 10.1016/j.healthpol.2022.07.012 PMC9356914

[31] Bakhit M, Baillie E, Krzyzaniak N, van Driel M, Clark J, Glasziou P, Del Mar C (2021) Antibiotic prescribing for acute infections in synchronous telehealth consultations: a systematic review and meta-analysis. BJGP Open. 5(6):BJGPO.2021.0106 10.3399/BJGPO.2021.0106 PMC9447298 34497096

[32] Norberg BL, Getz LO, Johnsen TM et al (2023) General practitioners’ experiences with potentials and pitfalls of video consultations in Norway during the COVID-19 lockdown: qualitative analysis of free-text survey answers. J Med Internet Res 25(1):e45812 36939814 10.2196/45812 PMC10131921

[33] Pogorzelska K, Marcinowicz L, Chlabicz S (2023) Understanding satisfaction and dissatisfaction of patients with telemedicine during the COVID-19 pandemic: an exploratory qualitative study in primary care. PLoS ONE 18(10):e0293089 37847684 10.1371/journal.pone.0293089 PMC10581451

[34] Jeffers H, Baker M (2016) Continuity of care: still important in modern-day general practice. Br J Gen Pract 66(649):396–397 27481958 10.3399/bjgp16X686185 PMC4979920

[35] DOH Ireland. Health in Ireland Key Trends 2023 [Internet]. 2024 Feb. Available from: https://www.gov.ie/pdf/?file=https://assets.gov.ie/285459/9f30e75f-c961-425f-80a9-92ed7bb279b1.pdf#page=null

[36] CDCGlobal. Global Measles Outbreaks [Internet]. Centers for Disease Control and Prevention. 2024 [cited 2024 Jan 24]. Available from: https://www.cdc.gov/globalhealth/measles/data/global-measles-outbreaks.html

[37] HSE. Covid-19 and cervical screening in Ireland: a coordinated approach to minimising harm [Internet]. Corporate. 2022 [cited 2024 Feb 11]. Available from: https://www2.healthservice.hse.ie/organisation/nss/news/covid19-cervical-screening-coordinated-approach-minimising-harm/

[38] Matenge S, Sturgiss E, Desborough J et al (2021) Ensuring the continuation of routine primary care during the COVID-19 pandemic: a review of the international literature. Fam Pract 39(4):747–761 10.1093/fampra/cmab115 PMC8515263 34611708

[39] Kroth PJ, Morioka-Douglas N, Veres S et al (2019) Association of electronic health record design and use factors with clinician stress and burnout. JAMA Netw Open 2(8):e199609 31418810 10.1001/jamanetworkopen.2019.9609 PMC6704736

[40] Sinsky C, Colligan L, Li L et al (2016) Allocation of physician time in ambulatory practice: a time and motion study in 4 specialties. Ann Intern Med 165(11):753–760 27595430 10.7326/M16-0961

[41] Arndt BG, Micek MA, Rule A et al (2024) More tethered to the EHR: EHR workload trends among academic primary care physicians, 2019–2023. Ann Fam Med 22(1):12–18 38253499 10.1370/afm.3047 PMC11233089

